# Renal Cell Carcinoma Masquerading as Acute Upper Gastrointestinal Bleeding

**DOI:** 10.1155/2024/7122744

**Published:** 2024-10-21

**Authors:** Aadesh Rayamajhi, Saurav Agrawal, Sandesh Rayamajhi, Manoj Lamsal, Dinesh Koirala

**Affiliations:** ^1^Maharajgunj Medical Campus, Tribhuvan University, Institute of Medicine, Kathmandu, Nepal; ^2^Nepalese Army Institute of Health Sciences College of Medicine, Kathmandu, Nepal; ^3^Department of Gastroenterology, Tribhuvan University Teaching Hospital, Kathmandu, Nepal

## Abstract

Acute upper gastrointestinal bleeding (UGIB) is a medical emergency with most common cause being peptic ulcer disease (PUD) or variceal bleeding. However, cancers that cause UGIB by invading the small intestine are uncommon, and the invasion of renal cell carcinoma (RCC) into the duodenum is an even rarer occurrence. A 55-year-old male presented with melena and right flank pain. Esophagogastroduodenoscopy (EGD) revealed an active bleeding source in the duodenum, later identified on contrast-enhanced computed tomography (CECT) as a duodenorenal fistula (DRF) caused by direct extension of the RCC. The imaging confirmed the presence of a malignant renal mass infiltrating adjacent organs. This atypical presentation of RCC with DRF manifesting as UGIB underscores the importance of comprehensive evaluations in cases of gastrointestinal bleeding to identify rare underlying causes.

## 1. Introduction

Acute upper gastrointestinal bleeding (UGIB) is a common medical emergency defined as hemorrhage originating from any part of the gastrointestinal (GI) tract proximal to the ligament of Treitz. UGIB typically presents as hematemesis in 40%–50% of cases, while 90%–98% of cases may present with either melena or hematochezia, the latter occurring in the context of a significant bleed. Common etiologies for UGIB include peptic ulcer disease (PUD), esophagitis, variceal bleeding, gastritis, and Mallory–Weiss tears [1–3].

Approximately 1%–4% of UGIB cases can be attributed to malignant causes, primarily due to malignancies of the esophagus and the stomach [4]. Tumors affecting the small intestine and causing UGIB are rare, constituting only 0.35% of all cancer cases, and their symptoms are often nonspecific [4, 5]. Even more uncommon are locoregional cancers that invade the duodenum and result in UGIB, typically linked to hepatobiliary and pancreatic cancers [5].

Primary renal cell carcinoma (RCC) presenting as GI bleeding due to direct extension is particularly rare, with only a few documented cases in the literature [6]. In such instances, RCC can invade the duodenum, leading to the formation of a duodenorenal fistula (DRF), which is an abnormal connection between the kidney and the duodenum. This fistula can result in UGIB, similar to an aortoduodenal fistula, due to rich vascularity of RCC [7].

The occurrence of GI bleeding in cases of primary RCC is infrequently documented in the existing literature [4]. Here, we report a case of acute UGIB from a DRF in a patient with RCC as the presenting complaint.

## 2. Case Presentation

A 55-year-old male with well-controlled systemic hypertension presented to the emergency department (ED) with complaints of right flank pain and multiple episodes of melena over two weeks. These symptoms were accompanied by loss of appetite, easy fatigability, and unintentional weight loss of 20 kg over six months. He also reported episodes of hematuria six months prior, which resolved spontaneously without medical intervention. The patient had a history of smoking and alcohol consumption, with no significant family history of malignant diseases.

Upon arrival at the ED, the patient was conscious and oriented. His vital sings included a pulse rate of 110 beats per minute, blood pressure of 90/60 mm Hg, respiratory rate of 20 breaths per minute, oxygen saturation of 96% on room air, and a fever of 101˚F. Physical examination revealed a pale, cachectic appearance and bilateral lower limb edema. Abdominal examination identified a palpable, firm mass measuring 5 × 6 cm in the right upper quadrant (RUQ). Examination of other systems was unremarkable.

Laboratory investigations were conducted upon admission, which revealed neutrophilic leukocytosis with a total leukocyte count of 38,000/mm^3^, neutrophils at 34,960/mm^3^ (92%), hemoglobin of 6.2 g/dL, and hematocrit of 20%. Coagulation profile, blood glucose, renal, and liver function tests were all within normal limits. Urinalysis showed no abnormalities, and peripheral blood film examination revealed normocytic normochromic red blood cells with neutrophilic leukocytosis.

**Figure 1:**
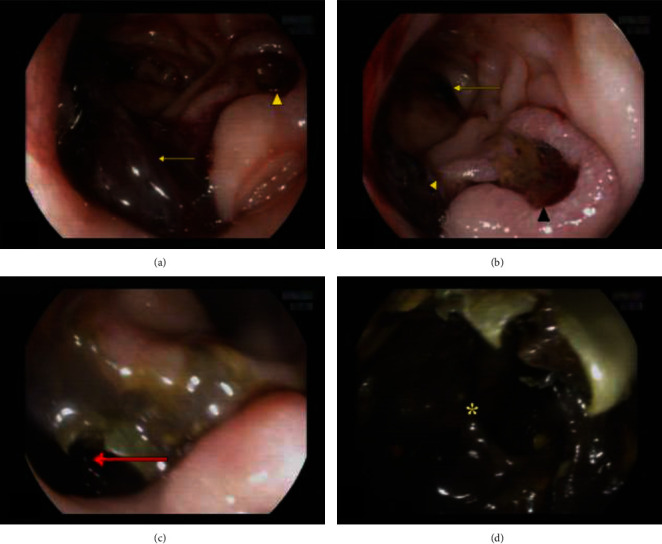
Esophagogastroduodenoscopy showing (a) large blood clot (arrow) and duodenal ulcer (arrowhead), (b) duodenal lumen (arrow), blood clot (yellow arrowhead), and a duodenal ulcer measuring approximately 1.5 cm with everted margin and adherent clot (black arrowhead), (c) an opening (red arrow) leading to renal cell carcinoma seen at D2 after removal of clot, and (d) a large cavity (asterisk) of necrotic renal cell carcinoma seen after entering the opening at D2.

Given the patient's hemodynamic instability, severe anemia, and signs of sepsis, immediate management was initiated. He received fluid resuscitation, blood transfusion, inotropes for hypovolemic shock along with bowel rest, proton pump inhibitors for UGIB, and empiric antibiotics for sepsis. After initial stabilization in the ED, an emergent EGD was performed. The EGD revealed no active bleeding in the stomach, but identified a duodenal ulcer approximately 1.5 cm with everted margin and adherent clot. Upon aspirating the clots, two openings distal to the first part of the duodenum were discovered. One led to the normal second part of the duodenum, while the other opened into a large cavity measuring approximately 8 × 5 cm, filled with blood clots and necrotic tissue (Figure 1). At this point, the nature and cause of this lesion were unclear.

Following the EGD, ultrasonography of the abdomen was performed, revealing a well-defined hetero-echoic lesion measuring 13 × 9 cm in the RUQ, with increased vascularity. The patient remained hemodynamically unstable, and due to his critical condition, he was transferred to the intensive care unit for closer monitoring.

Over the next few days, blood and urine cultures were obtained to further investigate the source of sepsis. While urine cultures showed no abnormalities, blood culture and sensitivity tests grew *Enterococcus faecium*, sensitive to teicoplanin. Intravenous meropenem, teicoplanin, and metronidazole were initiated for sepsis management.

**Figure 2:**
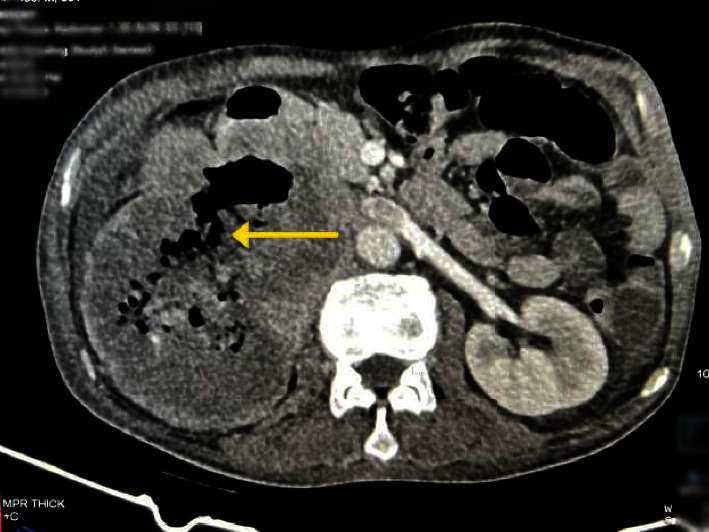
Contrast-enhanced computed tomography scan of axial section of abdomen showing duodenorenal fistula (arrow).

**Figure 3:**
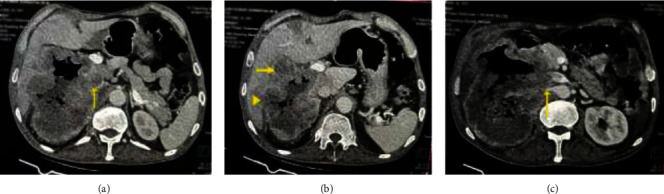
Contrast-enhanced computed tomography scan showing an RCC invading (a) inferior vena cava with thrombus (arrow), (b) fifth (arrow) and sixth segments (arrowhead) of the liver, and (c) right renal artery (arrow).

Once the patient's condition was sufficiently stabilized, a CECT scan of the abdomen was performed for further evaluation. The CECT scan revealed a large heterogeneously enhancing soft tissue density mass in the right renal fossa with fistulous communication to the second and third parts of the duodenum (Figure 2). The mass infiltrated segments five and six of the liver, the pancreatic head, and the right psoas muscle and showed inferior vena cava (IVC) thrombosis (Figure 3) confirming the presence of a malignant mass originating from the right kidney.

Consultations with GI surgery and urology recommended chemotherapy and palliative surgery. However, given the patient's frail state, the debulking surgery was rescheduled, to focus on his overall well-being. On day eight of admission, due to persistently lower hemoglobin level and melena, an interventional radiology consultation for the identification and embolization of the bleeding vessel was planned, but the patient refused and opted to continue his care at home due to financial constraints. He was hemodynamically stable at the time of discharge.

## 3. Discussion

Acute UGIB typically presents with symptoms such as fresh blood hematemesis, coffee ground emesis, and/or melena, with or without hemodynamic compromise. In most cases of acute UGIB, the primary causes are attributed to factors other than varices, accounting for a significant 80% to 90% of cases. Among these nonvariceal causes, the most common contributors include gastroduodenal peptic ulcer (20% to 50%), gastroduodenal erosions (8% to 15%), erosive esophagitis (5% to 15%), Mallory–Weiss tear (8% to 15%), arteriovenous malformations (5%), and other conditions such as Dieulafoy's lesion or upper GI tract malignancy [8]. Our patient had a history of melena for two weeks and presented to the ED with hemodynamic instability. Although PUD is a common cause of UGIB in our context, based on history and a palpable mass in RUQ, we initially suspected a bleeding upper GI tract malignancy. Following stabilization, EGD showed a duodenal ulcer and an abnormal opening that led to a large cavity, effectively ruling out esophageal and gastric malignancies. We could not differentiate the nature and etiology of lesion, and subsequently CECT abdomen was done.

RCC is the third most common malignancy of the urinary tract and has the potential to metastasize to various organs [9]. It often presents with a wide range of clinical manifestations and is typically diagnosed incidentally during investigations for nonspecific abdominal symptoms [7, 10]. The classic triad of hematuria, flank pain, and abdominal mass is seen in only 4%–17% of cases [10]. More than half of RCCs are detected incidentally with approximately 2% of patients presenting with locally advanced disease and about 26.7% diagnosed with stage two or three disease. Infiltration of neighboring organs such as the liver, duodenum, and pancreas by RCC is uncommon [11].

In our patient, a malignant right renal mass was discovered invading the pancreas, duodenum, and liver, with an IVC thrombus observed on the CECT scan. Due to the intimate proximity of the duodenum to the right kidney, it stands out as the most frequently affected site in GI tract involvement [9]. Additionally, the limited movement and absence of posterior peritoneal covering make the second part of the duodenum more vulnerable [12]. In cases of locally advanced renal cancer, such as in our patient, the tumor can infiltrate the second part of the duodenum, leading to the formation of a DRF.

While data on DRF are limited, the literature reveals an association between RCC and the renoalimentary fistulas, with colorenal fistulas being the most common subtype and DRF being extremely rare [12]. DRF typically occurs in the setting of chronic perinephric inflammation due to renal calculi or obstruction, but DRF secondary to RCC is uncommon and often unrecognized [13]. In our patient, the EGD revealed an abnormal opening in the second part of the duodenum leading to a cavity and the CECT scan confirmed this tract as DRF. Bleeding from the renal mass through this tract into the duodenum was responsible for the melena observed.

RCC can induce GI bleeding by directly invading tissues or metastasizing and invading nearby blood vessels [14]. Bleeding is more commonly seen with metastatic lesions within the GI tract or as a relapse occurring several years after undergoing nephrectomy for RCC [15]. UGIB has also been reported in a patient treated with microwave ablation for RCC [16]. However, GI bleeding from a DRF secondary to direct invasion as the initial presentation of RCC is not well-documented in the literature.

The patient's prognosis in cases of advanced RCC with DRF is typically guarded due to the aggressive nature of the malignancy and the complexity of the condition. Following his decision to opt for home care, the patient was advised on palliative care options to manage symptoms and maintain quality of life. Regular follow-up appointments were scheduled to monitor his condition, manage pain, and address any complications arising from the malignancy or the fistula. The importance of supportive care and a multidisciplinary approach involving oncology, palliative care, and primary care was emphasized to ensure the best possible outcomes for the patient.

## 4. Conclusion

Acute UGIB can be an uncommon presentation of RCC. When typical causes such as PUD, bleeding varices, and erosions have been excluded, sinister etiologies such as malignancy involving the GI tract have to be taken into consideration, especially in the presence of significant weight loss. In this case, the discovery of an infiltrative right malignant renal mass with a fistulous connection to the second part of the duodenum highlights the importance of conducting thorough investigations in evaluating patients with UGIB. This case emphasizes the necessity for clinicians to maintain a high index of suspicion for rare causes of UGIB and to pursue comprehensive evaluations to ensure accurate diagnosis.

## Data Availability

The data used to support the findings of this study are available from the corresponding author upon reasonable request.
